# Microfluidic enrichment of plasma cells improves treatment of multiple myeloma

**DOI:** 10.1002/1878-0261.12201

**Published:** 2018-05-12

**Authors:** Yunjing Zeng, Li Gao, Xiaoqing Luo, Yan Chen, Mustafa H. Kabeer, Xuelian Chen, Andres Stucky, William G. Loudon, Shengwen C. Li, Xi Zhang, Jiang F. Zhong

**Affiliations:** ^1^ Division of Periodontology, Diagnostic Sciences & Dental Hygiene Division of Biomedical Sciences Herman Ostrow School of Dentistry University of Southern California Los Angeles CA USA; ^2^ Department of Hematology Xinqiao Hospital Army Medical University Chongqing China; ^3^ Shenzhen Institutes of Advanced Technology Chinese Academy of Sciences Beijing China; ^4^ Children's Hospital of Orange County University of California‐Irvine School of Medicine Orange CA USA

**Keywords:** microfluidic, myeloma, risk stratification

## Abstract

Cytogenetic alterations form the basis for risk stratification for multiple myeloma (MM) and guide the selection of therapy; however, current pathology assays performed on bone marrow samples can produce false‐negatives due to the unpredictable distribution and rarity of MM cells. Here, we report on a microfluidic device used to facilitate CD45 depletion to enhance the detection of cytogenetic alterations in plasma cells (PCs). Bone marrow samples from 48 patients with MM were each divided into two aliquots. One aliquot was subjected to classic flow cytometry and fluorescent *in situ* hybridization (FISH). The other first went through CD45^+^ cell depletion, further enriched by microfluidic size selection. The enriched samples were then analyzed using flow cytometry and FISH and compared to those analyzed using the classic method only. Unlike the traditional method, the microfluidic device removed the CD45^+^ leukocytes and specifically selected PCs from the remaining white blood cells. Therefore, the microfluidic method (MF‐CD45‐TACs) significantly increased the percentage of CD38^+^/CD138^+^ cells to 37.7 ± 20.4% (*P* < 0.001) from 10.3 ± 8.5% in bone marrow. After the MF‐CD45‐TAC enrichment, the detection rate of IgH rearrangement, del(13q14), del(17p), and 1q21 gains, rose to 56.3% (*P* < 0.001), 37.5% (*P* < 0.001), 22.9% (*P* < 0.001), and 41.7% (*P* = 0.001), respectively; all rates of detection were significantly increased compared to the classically analyzed samples. In this clinical trial, this microfluidic‐assisted assay provided a precise detection of cytogenetic alterations in PCs and improved clinical outcomes.

**Abbreviations**
AJH: American Journal of Hematology
DLD: deterministic lateral displacement
FACS: fluorescence‐activated cell sorting
FISH: fluorescent *in situ* hybridization
IMWG: International Myeloma Working Group
MACS: magnetic‐activated cell sorting
MF‐CD45‐TACs: microfluidic–CD45 depletion–tetrameric antibody complexes
MM: multiple myeloma
PCs: plasma cells
PDMS: polydimethylsiloxane
TACs: tetrameric antibody complexes
VRD: bortezomib, lenalidomide, and dexamethasone
VTD: bortezomib, thalidomide, and dexamethasone

## 1 Introduction

Multiple myeloma (MM) is an incurable neoplasm of plasma cells (PCs) that affects more than 20 000 people annually in the United States. Risk stratification, primarily based on cytogenetic abnormalities, has emerged as essential for its management (Mikhael *et al*., 2013). Thalidomide, lenalidomide, and pomalidomide, which represent the first to third generations of immunomodulatory drugs, respectively, are used for MM maintenance therapy. Cytogenetic alterations form the basis of MM risk stratification and selection of immunomodulatory drugs for therapy (Nathwani *et al*., 2016). PCs undergo clonal evolution: In earlier and smoldering disease, CD45‐positive cells predominate, whereas CD45‐negative PCs are more prevalent in patients with advanced disease (both new and relapsed) (Kumar *et al*., 2005). Thus, both CD45^**−**^ and CD45^**+**^ can serve as prognostic biomarkers for MM (Gonsalves *et al*., 2016) but with distinct prognoses. The tumor load in patients with CD45^−^ cells is higher than those with CD45^+^ cells, which could be explained by the lower proliferation rate of the latter population (Asosingh *et al*., 2001). Malignant PCs manifest as CD45^−^ cells with co‐expression of CD38/CD138 plus CD19 and CD56, implying the clinical significance of CD45^−^ cells in risk stratification of MM (Langer *et al*., 2016). Therefore, depletion of CD45^+^ cells in bone marrow could enrich PCs for this purpose.

The rarity and sporadic distribution of PCs in bone marrow often leads to false‐negative results when fluorescence‐activated cell sorting (FACS) or cytogenetic detection is performed directly on a bone marrow biopsy sample. Also, morphologic heterogeneity and cytogenetic heterogeneity affect the flow cytometric recovery of PCs in bone marrow aspirates of patients with MM (Cogbill *et al*. 2015), resulting in inconsistent biomarker profiles (Kim *et al*. 2012) and affecting treatment decisions (Nadav *et al*. 2006). Improving the sensitivity and precision of MM diagnosis could therefore significantly impact clinical outcomes.

**Figure 1:**
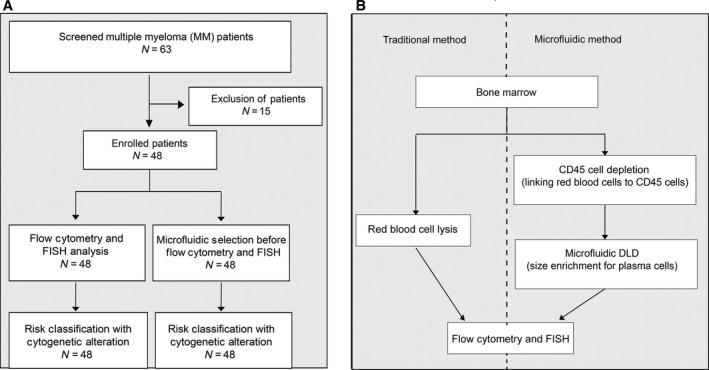
Schematic flowchart of study. (A) Consolidated Standards of Reporting Trials (CONSORT) diagram. A total of 63 patients were screened for eligibility. Only 48 patients were newly diagnosed with MM before receiving any treatment. These patients were enrolled, and their bone marrow obtained at diagnosis was divided into two aliquots: One aliquot underwent traditional flow cytometry and FISH analysis, and the other aliquot was subjected to microfluidic selection for enrichment of CD45‐PCs, then subjected to flow cytometry and FISH analysis. Results from both methods were compared. (B) Comparison of traditional method to microfluidic method (MF‐CD45‐TACs). MF‐CD45‐TACs significantly enrich PCs for flow cytometry and FISH assays and improve the accuracy of these assays.

Target cell enrichment could overcome the rarity and sporadic distribution of PCs in bone marrow. Density gradient centrifugation and magnetically labeled antibodies with magnetic‐activated cell sorting (MACS) have been widely used to enrich target cells in blood samples. MACS enrichment of CD138^+^ cells for fluorescent *in situ* hybridization (FISH) in MM diagnosis has been reported (Chen *et al*., 2007). However, MM cells with low levels of CD138 have also been associated with poor prognosis.(Kawano *et al*., 2012) Therefore, a better enrichment method is needed. Here, we report a novel microfluidic approach, combining microfluidic size selection and CD45 depletion with tetrameric antibody complexes (TACs) for enrichment of MM cells in bone marrow samples (MF‐CD45‐TAC enrichment). Our study showed that this approach significantly improves the detection of rare genetic alterations in PCs. Parallel diagnosis performed for 48 patients (Fig. 1) showed that the microfluidic enrichment approach represents a significant improvement over direct flow cytometry and FISH and leads to more precise diagnosis and prognosis. Implementation of this modified diagnostic assay in clinics could improve current clinical outcomes of MM.

## 2 Methods

### 2.1 Patient enrollment

We enrolled 48 newly diagnosed and untreated patients with MM in this study. BM samples of patients were obtained at diagnosis with informed consent. The sample from each patient was divided into two parts. One part was directly subjected to flow cytometry and FISH while the other part was processed with microfluidic deterministic lateral displacement (DLD) (McGrath *et al*., 2014) and CD45 depletion with TACs to link CD45^+^ cells with red blood cells (Z‐genetic medicine, USA). The CD45‐depleted (MF‐CD45‐TACs) cells were then subjected to the same FACS and FISH analysis as the classic assays. The expression of CD38 and CD138 (Ise *et al*., 2016; Langer *et al*., 2016; Matsue *et al*., 2016) was detected using flow cytometry (Chen *et al*., 2016; Wang *et al*., 2016). FISH analysis was used to reveal 13q deletion, 17p deletion, IgH rearrangement, and 1q21 gains (Rajkumar, 2016).

### 2.2 Microfluidic device fabrication

**Figure 2:**
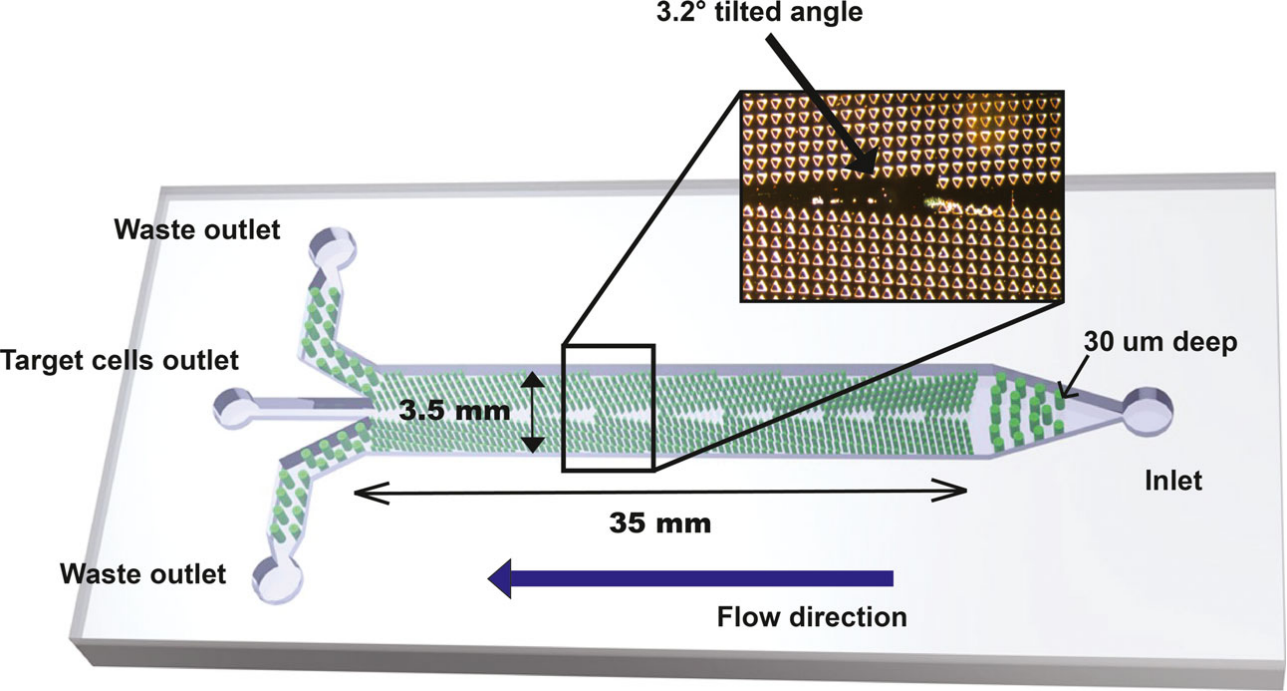
Schematic illustration of the microfluidic DLD device. The microfluidic DLD device consists of an inlet, three outlets, and a central flow chamber with micropost array. The flow chamber is 35 mm long, 3.5 mm wide, and 30 μm high. The micropost array of DLD chip is tilted at an angle of 3.2° relative to the fluid flow direction to facilitate cell separation.

Microfluidic DLD devices were fabricated with standard photolithography and soft lithography techniques. Negative photoresist SU8‐3025 (Microchem, Westborough, MA, USA) was used to fabricate the master mold on a silicon wafer with a photomask. The patterned silicon wafers were silanized with chlorotrimethylsilane (Aldrich, Burlington, MA, USA) to facilitate particle desorption mass spectrometry mold release. Polydimethylsiloxane (PDMS, RTV615; General Electric, USA) mixed with curing agent (5 : 1 w/w ratio) was poured into the silicon mold and cured at 80 °C for 1 h. Holes were punched for inlet and outlets, and the PDMS mold was bonded to glass slides after oxygen plasma treatment. The design of the DLD device is shown in Fig. 2.

### 2.3 Microfluidic enrichment of plasma cells (MF‐CD45‐TACs)

The microfluidic DLD design consisted of an inlet, three outlets, and a central flow chamber with micropost array, as shown in Fig. 2. The flow chamber was 35 mm long, 3.5 mm wide, and 30 μm high. The micropost array was tilted at an angle of 3.2° relative to the fluid flow direction. For each patient, a 1.0 mL sample of bone marrow fluid and 500 μL CD45 TACs were mixed at room temperature for 30 min. The mixture was slowly added into 2.0 mL Ficoll in a test tube. Low‐speed/low‐temperature centrifugation was performed at 450 ***g*** for 15 min. The white film layer at the interface of the plasma and the Ficoll solution was transferred to a fresh tube. Then, the white film layer (50–100 μL, 1 × 10^6^ cells) was loaded into DLD chip for PC enrichment. The DLD critical deflection diameter is designed such that large cells (PCs) flow in a bumping mode and thus are concentrated in the center of chamber, while small cells (erythrocytes and most of leukocytes) follow the mainstream direction. We optimized the design in the outlet portion to maximize the PC enrichment. The outlet portion of the device consists of a row of microposts with gradually decreasing gaps. With this design, although part of the erythrocytes and leukocytes escaped through the gaps at the outlet, the PCs can be fully collected once they were concentrated in the DLD chip. The resulting DLD‐enriched PCs were then subjected to FACS detection.

### 2.4 FACS analysis

Samples with or without microfluidic enrichment were incubated in 20 μL CD20‐PerCP, CD38‐APC, or CD138‐FITC, for 30 min in the dark at room temperature, and hemolysin was added for 5 min. After being washed with PBS twice, the supernatant was discarded upon centrifugation. All reagents were from the BD, USA, and analysis was performed on BD equipment: four‐color BD FACSCalibur or Six‐color BD FACS Canto II.

### 2.5 Bone marrow specimen collection and preparation

Two mililitre bone marrow was collected from each patient; 5–7 mL PBS was added to each sample and gently mixed. The mixture was centrifuged at 300 ***g*** for 10 min. Eight mililitre 0.075 m KCl solution preheated to 37 °C (to reduce permeability) was added to the cell pellets and mixed in a 37 °C hypotonic water bath for 30 min, by gently blowing on the suspended cells 2–3 times. For prefixture, 2 mL immobilized solution (methanol : glacial acetic acid at a ratio of 3 : 1) was slowly added to the hypo‐osmotic cellular mixture, which was gently pipetted up and down to make a cell suspension and centrifuged at 300 ***g*** for 10 min. To the supernatant, 6–8 mL fixative was added and mixed well. The mixture was allowed to stand for 10 min and then centrifuged at 300 ***g*** for 10 min; this step was repeated once. The cell suspension was prepared for slides with the appropriate amount of fixation liquid and placed at room temperature overnight, followed by baking at 56 °C for 20 min to 2 h, and then, the slides were dried and stored at room temperature.

### 2.6 Fluorescence *in situ* hybridization (FISH)

The slides prepared as described above were immersed in a 2× SSC (pH 7.0) solution for 30 min in a 37 °C water bath. The glass slides were then dehydrated in a series of 70% ethanol, 85% ethanol, and 100% ethanol for 2–3 min each with natural drying. The preparation of the probe was performed according to the manufacturer's manual. The preparation was centrifuged, vortexed and mixed, and again briefly centrifuged. The slide was naturally dried and then placed in a 56 °C oven to preheat for 5 min. Ten microlitre probe mixture was dropped onto the hybridization area of slide and immediately covered with the coverslip, followed by sealing the edges with rubber. Bone marrow samples were denatured at 75 °C for 5 min and hybridization conditions at 42 °C for 16 h. The well‐sealed slide was subjected to denaturation hybridization overnight with the hybridization instrument. The coverslip was removed, immediately placed in 50% formamide/2× SSC washing solution, washed twice, and rinsed for 10 min, with oscillation 1–3 s in 46 °C water baths. The slide was also washed with 2× SSC solutions and then rinsed for 10 min with oscillation 1–3 s in a 46 °C water bath. The glass slide was washed with 0.1% NP‐40/2× SSC solution and rinsed for 5 min, with oscillations of 1–3 s, in a 46 °C water bath. The glass slide was soaked in 70% ethanol at room temperature and rinsed for 3 min, then allowed to dry naturally. The dried glass slide was restained in the dark by adding 15 μL DAPI counterstain to the target area and immediately covering with the coverslip. The slide was allowed to rest in a dark place for 10–20 min before observations were made.

### 2.7 Statistical analysis

A paired‐samples *t*‐test was used for between‐group comparison of CD38^+^ CD138^+^ cells. Comparisons of 13q deletion, 17p deletion, IgH rearrangement, or 1q21 gains were evaluated with paired chi‐square tests. spss version 16.0 statistical software (IBM, Armonk, New York, NY, USA) was used.

## 3 Results

As an improvement over traditional methods, we developed a microfluidic method to enrich PCs for flow cytometry and FISH assays (Fig. 1). As schematized in Fig. 1, the microfluidic method (MF‐CD45‐TACs) significantly increased the percentage of CD38^+^/CD138^+^ cells to 37.7 ± 20.4% (*P* < 0.001) from 10.3 ± 8.5% in unmanipulated bone marrow (Table 1). This indicates that the percentage of PCs was increased by the depletion of CD45^+^ cells. The increase in PC concentration directly impacts the risk stratification of patients with MM. Before sorting, 11 patients (22.9%) showed evidence of rearrangements involving the 14q32 region, and deletion of 13q14 was found in six patients (12.5%). In addition, deletions of 17p and 1q21 gains were, respectively, detected in three (6.25%) and nine (18.8%) patients. After the MF‐CD45‐TAC enrichment, the percentage of detected IgH rearrangement, del(13q14), del(17p), and 1q21 gains, rose significantly, to 56.3% (*P* < 0.001 vs. classic analysis group), 37.5% (*P* < 0.001), 22.9% (*P* < 0.001), and 41.7% (*P* = 0.001), respectively. Thus, our microfluidic method (MF‐CD45‐TACs) can enrich PCs and significantly increase the detection rates of genetic aberration in patients with MM.

**Table 1:** Demographic and clinical characteristics of the patients.^a^

	Characteristic	Classic selection group (*N* = 48)	Microfluidic selection group (*N* = 48)
Age (year), no.	Median	60	60
	Range	41–78	41–78
Body weight (kg)	Median	61.5	61.5
	Range	42–75	42–76
Sex^b^, *n* (%)	Male	33 (69)	33 (69)
	Female	15 (31)	15 (31)
Diagnosis: Stage, *n* (%)	I	10 (20.8)	10 (20.8)
	II	14 (29.2)	14 (29.2)
	III	24 (50)	24 (50)
CD38^+^/CD138^+^, %^b^		10.3 ± 8.5	37.7 ± 20.4
Cytogenetic abnormality, *n* (%)	IgH	11 (22.9)	27 (56.3)
	13q14	6 (12.5)	18 (37.5)
	17p‐	3 (6.25)	11 (22.9)
	1q21	9 (18.8)	20 (41.7)
Disease risk, *n* (%)^c^ (FISH for risk stratification)	Low‐risk	5 (10)	9 (19)
	Intermediate‐risk	38 (80)	27 (56)
	High‐risk	5 (10)	12 (25)

a The chi‐square test was used for categorical variables, and analysis of variance.

b *P *< 0.001 for the difference in the distribution of characteristics among the two groups.

c Disease risk was categorized as low, intermediate, high, as previously described. (Rajkumar, 2016) *P *< 0.01 for the difference in the distribution of characteristics among the two groups.

### 3.1 Impact of MF‐CD45‐TACs in clinical outcomes

**Figure 3:**
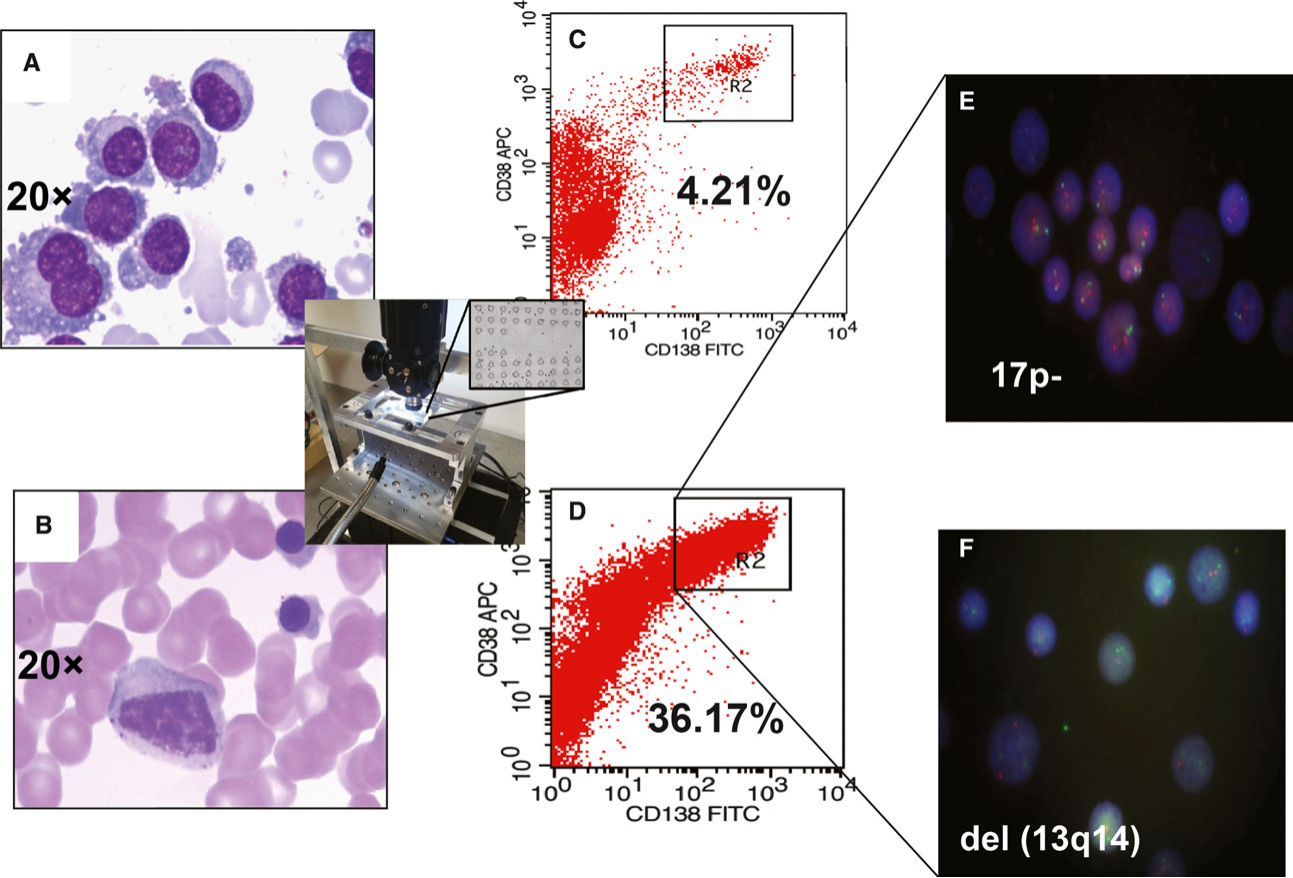
Improved clinical outcomes with microfluidic CD45 depletion (Patient 1). (A) bone marrow smear at the time of initial diagnosis. PC abnormalities increased, showing clustered or scattered distribution, with mainly mature and naive cells and similar body size. Coarse chromatin, rich cytoplasm, and tumorlike prominence were observed in the nucleus. (B) Bone marrow smear after effective treatment (complete remission). Active bone marrow hyperplasia, granulocyte, and erythroid cells were observed at all stages; the whole PCs were rare, with normal morphology and no typical abnormal PCs. (C) Flow cytometry without microfluidic enrichment. PCs (CD38^+^ CD138^+^) were only 4.21%, and no FISH cytogenetic abnormalities were found (low‐risk). (D) After microfluidic enrichment (center inset), PCs (CD38^+^/CD138^+^) increased to 36.17%. (E) FISH on enriched PCs showed 17p‐ (red: D13S319; green: P53). (F) FISH showed del(13q14) in enriched PCs (red: D13S319; green: RB1). The patient was reclassified and treated as having high‐risk MM. Treatment led to complete remission.

In the clinic, MF‐CD45‐TAC technology has demonstrated that it can improve diagnosis and change treatment. Based on the International Myeloma Working Group, bortezomib and carfilzomib improve complete response, progression‐free survival, and overall survival in t(4;14) and del(17/17p), whereas lenalidomide may be associated with improved progression‐free survival in t(4;14) and del(17/17p) (Sonneveld *et al*., 2016). Therefore, different cytogenetic alterations require different treatment plan. Patient 1, a 60‐year‐old male, was diagnosed with MM, type (IgG, λ), ISS stage III period. No FISH cytogenetic abnormalities were found using the classic method, leading to low‐risk stratification; however, upon MF‐CD45‐TAC enrichment, PCs showed deletion(17p‐) and del(13q14) (Fig. 3), leading to reclassification as high risk. This diagnosis changed the treatment plan from the initial VTD (bortezomib, thalidomide, and dexamethasone) to VRD (bortezomib, lenalidomide, and dexamethasone) induction therapy, and this protocol achieved very good partial remission. After four courses of autologous stem cell transplantation, the patient showed complete remission. After another five courses of VRD consolidation therapy, the patient's condition continues to be stable in complete remission with maintenance RD therapy.

**Figure 4:**
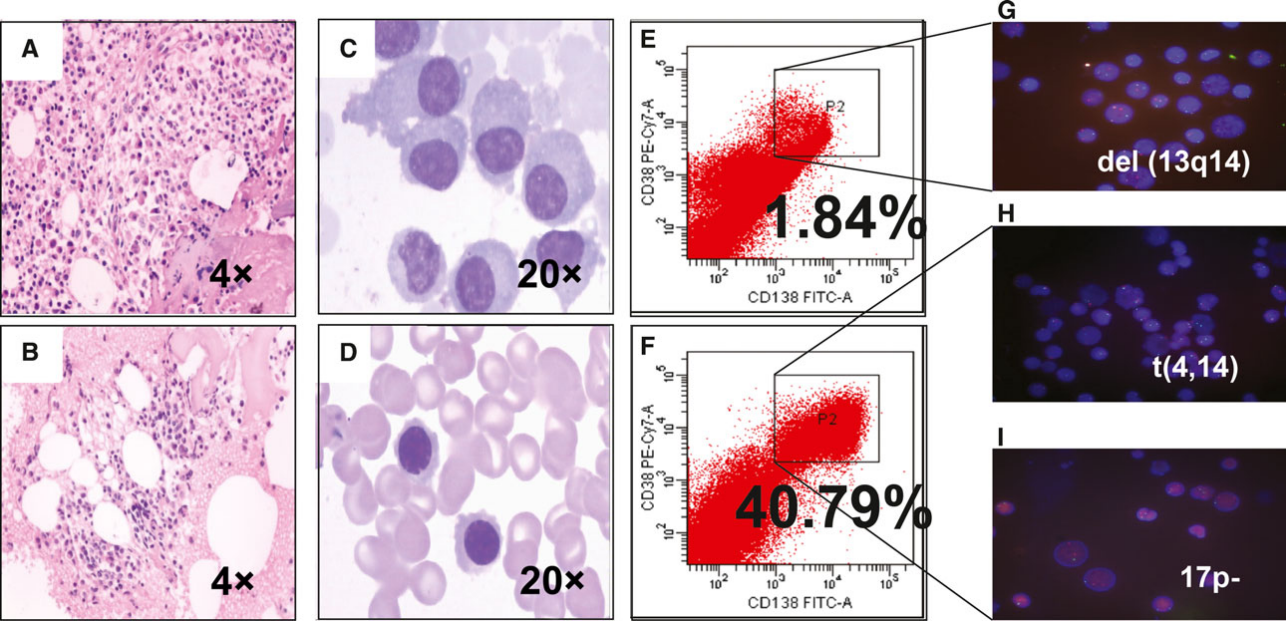
Microfluidic risk stratification improves clinical outcomes of MM (Patient 2). (A) Bone marrow at diagnosis. Active granulocyte hyperplasia, no precursor cell cluster distribution phenomenon, and late cell‐stage cells were observed: hematopoietic tissue (85%) and adipose tissue (15%). The erythrocytes were hyperplastic with erythrocyte clusters. No macular proliferation was observed (4×). (B) Partial remission was achieved with revised risk stratification. The proliferation of bone marrow hematopoietic tissue, the adipose tissue hyperplasia, and the granulocyte hyperplasia all were active. No progenitor cells were clustered. Cells in the later stages were visible. The erythrocytes proliferated and were not seen in the trabecular region. Megakaryocyte proliferation increased with morphology and distribution of roughly normal (4×). (C) At diagnosis, bone marrow PC abnormalities included clustered and scattered distribution of primitive and immature PCs, with large cell body, fine chromatin, visible nucleolus, and abundant cytoplasm (20×). (D) After treatment for high‐risk MM, PCs were rare and had normal morphology; no typical abnormal PCs were observed (20×). (E) At diagnosis without microfluidic enrichment, PCs (CD38^+^/CD138^+^) were only 1.84%. (F) After microfluidic enrichment, PCs (CD38^+^/CD138^+^) increased to 40.79%. (G) Without microfluidic enrichment, FISH showed IgH rearrangement and del(13q14), leading to classification as intermediate risk (red: D13S319; green: P53). (H, I) After microfluidic enrichment, in addition to del(13q14), FISH showed t(4,14) fusion (yellow dots) and 17p‐ (red: D13S319; green: P53), patient was reclassified and treated as high risk, which led to efficacious treatment.

Patient 2, a 41‐year‐old male, was diagnosed with MM, (IgG, λ) type, ISS stage III period. FISH analysis without microfluidic enrichment showed del(13q14) and IgH rearrangement. After MF‐CD45‐TAC enrichment, FISH analysis showed not only these two cytogenetic abnormalities, but also del(17p), leading to a shift from intermediate‐risk to high‐risk stratification (Fig. 4). After the patient received four cycles of VTD regimen and reached very good partial remission, his brother was found to be compatible through human leukocyte antigen typing and donated allogeneic hematopoietic stem cells for transplantation. This combination of therapies helped the patient achieve stringent complete response upon receiving thalidomide maintenance therapy. If we had not found del(17p), treatment would have instead involved iso‐allogeneic stem cell transplantation.

These two cases provide evidence that our microfluidic‐based MF‐CD45‐TACs can be used to improve diagnosis, risk stratification, and management of patients with MM for better treatment outcomes. In light of literature showing that genetic profiling is an important factor affecting a variety of treatment outcomes, we have developed a rapid, simple, and sensitive assay for clinical applications.

## 4 Discussion

Classic diagnosis of MM requires at least 10% of the cells in bone marrow to be PCs or a biopsy‐proven plasmacytoma plus evidence of one or more MM defining events (Rajkumar, 2016). Molecular classification shows that MM is a highly heterogeneous PC population with several different cytogenetically distinct PC malignancies, including gain(1q), del(1p), del(17p), del(13), RAS mutations, and secondary translocations involving Myc – all of which can influence disease course, response to therapy, and prognosis (Rajkumar, 2016). Accurate risk stratification, as determined by cytogenetic abnormalities, is considered to be the most important prognostic factor essential to guiding individual treatment of MM (Kapoor *et al*., 2010). Despite existing guidelines recommending close observation until progression to symptomatic disease, clinical studies have confirmed that early treatment can improve overall survival rates for high‐risk patients. Cytogenetics and molecular genetics can help in the early assessment of risk; thus, more sensitive and accurate screening methods like ours may benefit these patients.

Although cytogenetic abnormalities can be identified by fluorescence *in situ* hybridization (FISH), the rarity and uneven distribution of myeloma cells in the bone marrow often results in high false‐negative rates. MACS is recommended to enrich myeloma PCs (Fonseca *et al*., 2009), but is expensive, requires a large volume of bone marrow, and is a time‐consuming and laborious procedure, resulting in inconsistency (Chen *et al*., 2007). Here, we provide a microfluidic MF‐CD45‐TAC enrichment scheme, allowing rapid enrichment of PCs, thereby improving accuracy of risk stratification through more sensitive cytogenetic detection, improving patient prognosis.

Our study resonates with the established AJH and IMWG criteria: patients with del(17p), t(14;16), and t(14;20) have high‐risk MM (Rajkumar, 2016). Patients with t(4;14) translocation and gain(1q) have intermediate risk. All others are considered standard risk. Initial treatment for those not in the high‐risk group consists of bortezomib, lenalidomide, and dexamethasone (VRD). In high‐risk patients, carfilzomib, lenalidomide, and dexamethasone is an alternative to VRD. This initial therapy is followed by autologous stem cell transplantation (Ludwig *et al*., 2013).

In this study, we used MF‐CD45‐TACs to improve the detection rate of cytogenetic abnormalities in newly diagnosed patients with MM. As demonstrated by the two example cases showed above, MF‐CD45‐TACs can improve risk stratification of MM and impact clinical outcomes. Thus, we provide a novel, sensitive assay for diagnosis, risk stratification, and management of patients with MM. Further large‐scale clinical randomized controlled trials are needed to determine whether the change in clinical treatment regimens can lead to prolonged overall survival and improved quality of life.

## Author contributions

SCL, XZ, and JFZ designed research studies; LG, YZ, XL, YC, XC, and AS helped in conducting experiments, acquiring, and analyzing data; LG, YZ, XL, YC, MHK, XC, AS, WGL, SCL, XZ, and JFZ contributed to writing the manuscript.
