# Corrigendum to: Microfluidic enrichment of plasma cells improves treatment of multiple myeloma

**DOI:** 10.1002/1878-0261.12874

**Published:** 2021-01-04

**Authors:** Yunjing Zeng, Li Gao, Xiaoqing Luo, Yan Chen, Mustafa H. Kabeer, Xuelian Chen, Andres Stucky, William G. Loudon, Shengwen C. Li, Xi Zhang, Jiang F. Zhong

The following statement has been added to the subsection 2.1 of Materials and methods of the paper by Zeng *et al*.^1^:

‘The study is approved by the Chinese Ethics Committee of Registering Clinical Trials (ID: ChiECRCT‐20160046) and conformed to the standards set by the Declaration of Helsinki’.
